# Supplementary material for: Are carbon emissions trading and green financial instruments synergistic? -Comprehensive quantitative research based on content analysis
Source: PLoS One. 2024 Mar 7;19(3):e0298601. doi: 10.1371/journal.pone.0298601 (PMC10919601; doi:10.1371/journal.pone.0298601)
Supplement: S1 Data — (ZIP) [file pone.0298601.s001.zip › Data/地级市Z_F工作报告.zip!/║■▒▒/╩«╤▀╩╨/└┤╫╘╣1⁄2╓┌║┼í╛┬φ┐╦╩2╛▌═°í┐_2015.docx]

各位代表：

现在，我代表市人民政府向大会作工作报告，请予审议，并请各位政协委员和其他列席会议的同志提出意见。

　一、2014年工作回顾

　　2014年，是竞进有为的一年。面对经济下行压力加大、改革发展和生态建设任务繁重的形势，全市人民从容应对挑战，奋力克难攻坚，圆满完成了年初既定的各项任务，顺利实现了经济升级增效、民生不断改善、生态持续优化、调水顺利实现、社会和谐稳定的目标，经济社会发展取得可喜成就。

　　（一）经济运行质效得到新提升

　　实现地区生产总值1200.8亿元，增长9.5%（现价增长11.1%）；地方公共财政预算收入85.8亿元，增长16.6%。

——农村经济稳中向好。实现农业增加值151.2亿元，增长5.1%。完成农产品加工产值280亿元，增长40%。粮食总产23.6亿斤，增长1.6%。新增特色产业基地34万亩。丹江口水产生态养殖基地列为国家综合标准化示范区，十堰（长岭）农产品加工园晋升为省级示范园。新增国家地理标志商标7件、“三品一标”产品30个。新增农村土地流转面积15.6万亩，培育家庭农场141家、农民专业合作社205家。完成小农水项目9个，除险加固病险水库60座，解决农村安全饮水22.3万人。实施库区移土培肥工程7.3万亩，配套坡改梯6万亩。改造和新修县乡村道1028.5公里、危房8392户。改造 “低电压”居民2.1万户。

——工业经济持续增长。完成规上工业总产值1903.3亿元，增长10.6%。工业利润增长7.1%，高于全省平均水平1.2个百分点。东风与沃尔沃合资公司投入运营，东风特种商用车有限公司正式成立。东风动力总成、装备、特商、零部件工业园及小康发动机等项目快速推进。国家汽车零部件质检中心基本建成。生物医药等5个产业集群被确定为全省重点成长型产业集群。落实工业项目零收费政策，搭建工业项目金融超市平台，累计担保总额超过40亿元。在四板市场新增上市企业26家。

——现代服务业加快成长。旅游业快速发展，新增4A级景区4家，新开通汉江水上游线路，成为全省智慧旅游试点城市。全年接待游客3435.4万人次，实现旅游收入242.7亿元，分别增长18%、20.1%。其中，武当山接待游客569.3万人次，实现旅游收入31亿元，分别增长16.2%、18.8%。万达广场、华西国际农商城、林安物流园、昌升国际商贸城等项目加速推进，快递产业园建成运行。培育亿元商贸物流企业16家，新建改扩建市场、便民超市、农家店449个，“武当珍品汇”在全国布点48家。电子商务产业孵化基地挂牌，丹江口市列入全国电子商务进农村试点，郧西县涧池乡下营村成为湖北唯一的全国淘宝村。养老服务业取得明显进展，培训基地建成使用，兴办社区养老服务中心45个。社会消费品零售总额548.7亿元，增长13.8%。

——发展后劲明显增强。全社会固定资产投资完成1112.3亿元，增长21.5%。重大项目快速推进，130个市级和14个省级重点项目超额完成年度计划。武当山机场建设全面铺开，武十城际铁路即将开工，谷竹、十房、郧十高速建成通车，京能热电项目核准开工，龙背湾水电站下闸蓄水，夹河关、孤山水电站前期工作基本完成。市场主体发展迅猛，新增市场主体7.1万户、规上工业企业196家（居全省第一）、限上商贸企业258家、市级农业龙头企业55家、高新技术企业31家。新增湖北著名商标35件、湖北名牌22个。新增贷款157.8亿元，增长21.8%。

　　（二）“一核多支点”建设开创新局面

　　——城乡建设同步加快。修定完成十堰市土地利用、城市总规，控制性详规和生态滨江新区规划，编制完成县乡村规划1162项，基本实现城乡规划全覆盖。全力推进市政重点工程建设，发展大道、龙门大道、风神大道二期、京东路、天马大道、长沙一级路建成通车，武当路、郧阳路、镜潭路改造完成，火车站北广场、西城大道、林荫大道二期、北环路、机场东路、清潭路、源园路改造全面开工。22条背街小巷、10条市政道路微循环项目改造完成，压缩天然气加气母站建成运营。四方山生态公园加快建设，顺利通过第三次国家园林城市评审。汉江生态经济带7大园区骨架基本形成，武当花谷、子胥湖新区、安阳绿谷等片区粗具规模，环库生态公路全线开工，丹江口市东环路建成通车，凉习路、柳五路等路段加快建设，建成库周生态隔离带23.6万亩。竹房城镇带新启动建设项目664个，城镇面积扩大10平方公里。

——城乡经济协调发展。“一核”发育良好，张湾区强力发展工业，东风动力总成等五大板块、三环工业园、生物产业园等重大项目投产见效；茅箭区加快发展现代服务业，第三产业占GDP比重达58.4%；郧阳区培育壮大特色产业，工业增加值增长23.1%，增速居全市第一；十堰经济技术开发区大力发展创新型产业集群，实现高新技术产值399.5亿元、增长20.1%。“多支点”竞相成长，丹江口市致力调整结构，转型发展成效明显；郧西县深度挖掘资源，文化旅游产业成为支柱产业；房县突破性发展八大产业，经济实力不断提升；竹山县打造经济升级版，生态产业占GDP比重达80%；竹溪县坚持绿色发展，五大产业特色鲜明。全市县域GDP511.3亿元，增速高于全市0.3个百分点。城镇常住居民人均可支配收入22143元，增长9.7%；农村常住居民人均可支配收入7046元，增长13.4%。

　　（三）全面深化改革实现新突破

　　——各项改革推进有力。调整优化行政区划，郧阳区正式成立。加快政府职能转变，行政审批项目由879项减少到272项，减幅达69%；全面清理和公开行政权力清单，部门行政权力和服务事项减少27%，成为全省行政审批最少的城市之一。农村土地制度、经营机制、投融资等各项改革扎实推进。卫生计生机构改革和食品药品监管体制改革顺利完成。创新财政资金管理方式，郧阳区统筹整合使用涉农资金经验在全省推广。多层次资本市场加快建设，湖北汽车及零部件交易中心和武汉股权托管交易十堰分中心获得批准，成功发行三期城投债，汉口银行入驻十堰。市及各县市区均获信用县市称号。房县、郧西县纳入全省第二批县级公立医院综合改革试点，民营医院增加到17家。

——对外开放成效斐然。加大招商引资力度，引进市外境内项目564个，到资362亿元，增长33.7%，与国药控股、山东高速等一批知名企业达成合作协议。加强与北京市对口协作，年度24个援助项目全部完成、37个合作项目基本完成。筹建十堰中关村科技成果产业化基地，7个项目落地。新增进出口经营权企业94家，外贸出口5.4亿美元，增长21.3%；实际利用外资1.8亿美元，增长15.6%。推进大通关建设，启动国家级出口汽车及零部件质量安全示范区创建工作，编制完成航空口岸建设规划，推进公路口岸、海关监管区、检验检疫区建设。

　（四）美丽十堰建设取得新进展

　　——生态建设推进扎实。十堰成为全国首批生态文明先行示范区，深入开展“五城联创”，创建国家级生态乡镇6个、省级生态村38个。《丹库 “十二五”规划》283个项目全部开工，完工229个。组织开展“清水行动”、“保水质、迎调水”百日攻坚、“向三大污染宣战”等行动，关闭不达标企业31家。五河治理实现了阶段性治理目标。南水北调中线工程正式通水，12月12日定为十堰生态文明日。治理水土流失面积161.3平方公里，植树造林30.2万亩，修复裸露山体7189亩，荣获省级森林城市称号。张湾黄龙滩、房县古南河晋升为国家级湿地公园。

——环境保护成绩实在。全省第一个全域环境监测站通过国家标准化验收，率先成立环境应急中心，率先形成PM2.5空气质量监测能力，率先启动机动车排气检测。主要污染物总量减排任务圆满完成，万元生产总值综合能耗下降9%，降幅居全省第一。全市地表水质量总体良好，城区空气质量优良天数达322天。

　　（五）保障和改善民生展现新作为

　　——改善民生办实事。持续加大民生投入，民生支出达210.7亿元，占地方公共财政预算支出达81.6%。新增就业5.4万人，五项社会保险参保人数达215万人次。保障城乡低保、五保对象35.1万人、新增养老床位3612张。新农合参合率达99.6%，医保参保率达98%。开工建设保障性住房4.97万套，建成2.3万套。物价保持稳定，居民消费价格上涨1.5%。实施以“结穷亲、帮穷户、拔穷根”为主题的精准扶贫工程，完成65个重点村整村推进，搬迁3900户，减少贫困人口9万人。张湾区、茅箭区比照享受幕阜山片区扶贫政策。十件实事年度任务全面完成。

——发展社会事业出实招。大力推进科技创新，再获全省科技创新先进市称号。加快实施“3331人才工程”，组织85名企业家赴浙江大学培训，遴选资助基层人才280名，培训基层骨干150名。着力发展教育事业，新建改扩建中小学172所、幼儿园108所，重庆路小学、阳光小学、龙门小学、北京路中学、柳林中学、车城高中、十堰高级职校建设有序推进，武当山国际武术学院正式招生，郧阳区、茅箭区分别通过国家义务教育均衡发展区验收和获省示范区称号。3个项目入选国家非物质文化遗产名录，13处文物点列入第六批湖北省文物保护单位。安装地面卫星接收设施3.5万套，提前完成广播电视村村通规划。新建城乡体育健身工程197个，参加省第十四届运动会取得佳绩。太和医院、人民医院、东风公司总医院、市疾控中心重点项目全面开工建设。创建健康城市通过省验收，计生工作获省政府表彰。

　　（六）民主法治建设结出新成果

　　——民主法治深入推进。自觉接受人大、政协监督，定期向人大报告工作、向政协通报情况，办理人大代表意见建议126件，办结率达100%，满意和基本满意率98.5%；代表走访选民建议91件、政协提案361件，办结率、满意和基本满意率均达100%。强化审计监督，项目管理水平和资金使用效益逐步提高，市审计局荣获全国审计机关先进集体称号。加强廉政建设，反腐倡廉保持高压态势。推进“法律六进”活动，十堰荣获全国“六五”普法中期先进城市称号。第九届村委会换届选举圆满完成。茅箭区蝉联全国和谐社区建设示范城区称号。国防动员、民兵预备役和拥军优属工作不断加强。

——文明创建成果丰硕。第四届全国文明城市提名通过复查，成功蝉联省级文明城市七连冠。丹江口市、竹山县、竹溪县、郧阳区荣获省级文明城市称号，丹江口市六里坪镇等41个乡镇（村）荣获省级文明乡镇（村）称号。丹江口市李国刚当选“全国十大最美村官”，竹山县王耀林、郧阳区李茂清当选“中国好人”，郧阳区外迁移民赵久富入列央视“感动中国”候选人。

——社会治理不断加强。纵深推进网格化建设，全面落实“一区一警、一格一员、一街一队”等防控机制，建立健全立体化社会治安防控体系。刑事案件下降15%，群体性事件下降25.8%，群众安全感测评位居全省前列。排查化解各类矛盾纠纷8871起，赴省、赴京上访总量下降，信访形势明显好转。安全生产形势稳定向好，四项指标大幅下降。大力整顿规范食品药品市场秩序，食品药品安全风险防控有效。

——工作作风持续转变。深入开展党的群众路线教育实践活动，严格执行中央“八项规定”、省委“六条意见”和市委“实施意见”，持之以恒纠正“四风”，政府自身建设得到加强。

全市民族宗教、外事、侨务、对台、气象、地震、人防、残联、老龄、统计、社科、地方志等工作都取得了新成绩。

各位代表！这是一份付出艰辛、得之不易的成绩单！归功于市委的坚强领导，归功于市人大、市政协的有效监督，归功于社会各界的鼎力支持，凝聚着全市人民的智慧和心血。在此，我代表市人民政府，向全市广大工人农民、知识分子、干部职工，向驻堰人民解放军、武警官兵，向各民主党派、工商联、人民团体和社会各界人士，表示衷心的感谢！

在总结成绩的同时，我们更清醒地认识到存在的问题：经济总量不大与质量不高并存，转型升级的要求十分迫切；三次产业协调增长不够与融合度不紧并存，结构调整任务艰巨；农村基础脆弱与城镇化发展不快并存，城乡统筹任重道远；民生保障能力不足与社会治理压力加大并存，离广大群众的期盼差距较大。尤其是当前经济下行压力大，实体经济面临困难多，而部分公务人员对新形势、新要求不适应，存在不愿作为、不敢作为、不会作为的现象，还有一些地方和部门腐败问题仍然严重。对这些问题，政府将先从自身找原因，增强忧患意识，敢于担当，毫不懈怠，扎实有效解决问题，决不辜负人民的厚望。#PAGE_BREAK#

　　二、2015年工作安排

　　今年是全面深化改革的关键之年，是全面推进依法治国的开局之年，也是全面完成“十二五”规划的收官之年。做好今年的工作，必须准确把握新常态的新要求，找准工作着力点和突破口，在抢抓机遇中把握工作主动权。

新常态之新，在于速度换挡，结构优化，动力转换，其实质是走质量效益型集约增长的新路子，实现更高质量、更有效率、更可持续的科学发展。十堰产业结构相对不优，新兴产业发展不足，生态保护要求至高，转型升级的任务十分艰巨，面临的压力、困难和挑战极为集中，对此我们务必要有足够的认识。同时更要看到，我们正面临一系列重大历史机遇和难得的发展条件：一是中央重点实施“一带一路”、长江经济带、京津冀协同发展三大战略，提升了十堰承东启西的战略区位功能，为十堰提供了更为广阔的发展空间。二是国家全面深化改革，全面推进依法治国，实施积极的财政政策和稳健的货币政策，实施消费拉动、创新驱动战略，将更好地发挥法治对改革、发展、稳定的引领和保障作用，促进改革红利、内需潜力、市场活力的释放。三是省委、省政府深入实施“一元多层次”战略，纵深推进生态文明建设、新型城镇化和秦巴山片区扶贫开发，必将为十堰建设和发展提供更加有力的支撑。四是东风与沃尔沃战略联盟形成、东风特种商用车有限公司运营、与北京对口协作纵深推进，必将加快十堰大开发、大建设、大发展。五是随着武当山机场、武十城铁、京能热电等一批重大项目强力推进，四条高速公路全面建成，必将为十堰建设区域性中心城市注入新的动力和活力。六是通过党的群众路线教育实践活动开展，全市广大干部精神状态好、干事劲头足、工作作风实，为我们战胜困难夺取胜利提供了良好的基础。

面对经济发展的新常态，只要我们顺应大势，遵循规律，不屈不挠，不馁不骄，以竞进向上的精神状态迎接新常态，以转型跨越的积极作为适应新常态，以改革创新的勇气引领新常态，就一定能克服前进道路上的任何艰难险阻，谱写新常态下十堰发展更加辉煌的新篇章。

今年政府工作的总体要求是：认真贯彻党的十八大、十八届三中、四中全会、中央经济工作会议和省委十届五次全会暨经济工作会议精神，紧紧围绕加快建设区域性中心城市和“两个走在前列”，以提高经济发展质量和效益为中心，主动适应经济发展新常态，突出创新驱动，全面深化改革，推进依法治市，着力稳增长、调结构、转方式、强支撑、保生态、惠民生，全面完成“十二五”目标任务，加快构建“一核多支点”发展格局，努力实现“经济发展量增质优、基础设施攻坚提速、生态水质持续优化、扶贫民生精准发力、法治社会和谐有序、党风政风清正廉明”六大目标。

经济社会发展的主要预期目标是：地区生产总值增长9.5%；地方公共财政预算收入增长12%；城镇常住居民人均可支配收入增长10%以上，农村常住居民人均可支配收入增长12%以上；城镇登记失业率控制在4.5%以内；居民消费价格指数和节能减排控制在省定目标以内。今年，将重点做好以下工作：

（一）全面深化改革，激发内生动力

　　改革是发展的制胜法宝。要以处理好政府和市场关系为核心，以深化行政审批制度改革为突破口，协调推进各领域改革，最大限度激发市场和社会活力，使改革新红利转化为发展新动能。

——坚决推进简政放权、放管结合的改革。全面取消非行政许可审批，大幅度减少前置审批；规范行政审批管理，落实市场准入负面清单，推广网上并联审批，开展好局长进大厅活动，提供高效便捷的行政审批和政务服务，政府既要从越位点退出，又要把缺位点补上。积极创新行政管理制度，完善事中、事后监管，做到法无授权不可为，法定责任必须为。进一步清理和规范中介服务，清除和部门捆绑的中介机构，推行中介服务项目目录管理，取消无法律规定、自行设立的服务项目；大力培育中介服务市场，优先培育具备多项资质条件的综合性中介服务机构。

——落实财税金融体制改革。深化预算改革，实行全面规范、公开透明的预算管理制度，所有使用财政资金的部门除涉密信息外都必须公开预决算。落实国家税改任务，扩大营改增领域。

——稳妥推进农村综合改革。积极开展确权颁证，充分激活农村土地、资产、劳力等资源，创新土地流转方式，完善土地流转服务机制，加快构建新型农业经营体系，促进家庭经营、集体经营、合作经营、企业经营等共同发展。

（二）深入促进开放，释放发展活力

　　开放与改革相伴而生、相互促进。要全方位扩大对内对外开放，加快培育竞争新优势，以开放促改革、促创新、促发展。

——全面放活市场。发挥市场对资源配置的决定性作用，坚持权利公平、机会公平、规则公平，增强各类所有制经济活力，为民间资本提供大显身手的平台。全面放开创业领域、放低创业门槛、放活创业方式，让更多的人以更低的成本、更广的途径实现创业梦想。积极营造创业崇高、致富光荣的社会风尚，让创业之风劲吹，让创业潮流涌动。放手发展各类市场主体，下大力气解决好融资、用地等难题，切实为市场主体发展保驾护航，营造大众创业、万众创新的良好环境，让所有市场主体活力充分释放。

——支持实体经济。加强要素资源的有效配置，优先保障重点产业、重点企业的需求；加大对中小微企业的支持力度，促其做实做强；创新政银企合作模式，促进金融更好地服务实体经济，争取新增贷款170亿元以上；继续坚持并发挥好首席服务官制度的作用，为企业更多、更快、更实地排忧解难。

——大力招商引资。强化招商引资工作首位度，整合招商资源，加强招商力量，提升招商质效。以长三角、珠三角、京津冀地区为主战场，引进一批科技型、创新型、环保型企业落户十堰，补充一批龙头型、补链型的项目和企业，提高十堰产业的配套性，拉长产业链条。加大招商项目服务力度，切实提高协议履约率、项目开工率、资金到位率，确保全年到位资金460亿元。

——提高经济外向度。围绕汽车、特色农业等优势产业产品，利用国内外知名展会平台，扩大内外贸易，不断开拓国内国际两个市场。推进国家级汽车及零部件质量安全示范区创建工作，完成十堰公路口岸和海关特殊监管区建设，申报验收保税仓库，启动铁路口岸建设，力争外贸出口突破6亿美元。

——推进区域协作。深入开展与北京对口协作，建立对口协作产业发展基金，加快推进市场对接。打破行政壁垒，消除区域、层级、部门、所有制制约，推进区域全方位合作、抱团发展，支持鼓励市内跨行政区域共建产业园区和经济协作。

　（三）转变发展方式，提升经济实力

　　创新驱动是培育增长动力和竞争优势的根本和关键，壮大支柱产业则是基础和前提。必须通过转型升级、调整结构来推动三次产业协调健康成长。

——发挥“牛鼻子”作用，推进新型工业化。坚持做大总量、优化结构、提升质效、两化融合，培育壮大骨干企业，加快推进转型升级，着力形成一主多元、多极支撑的工业发展格局。一是壮大规模。锁定千亿级汽车工业、百亿级特色工业、百亿级园区工业发展目标，围绕企业成长、园区集聚、产业集群三大重点，突出抓好企业进规、培强、满园等工作，推进倍增计划，新增规上工业企业100家以上，实现工业总产值2200亿元以上。二是优化结构。用心用情用力服务东风，扎实推进“整车专用化”战略，重点支持东风商用车动力总成变速箱、东风特商猛士民品、东风小康30万台微型发动机、驰田3万台高轻自卸车扩能等项目尽快建成投产，加快培植大运、海龙、神河等专用车骨干企业。在优化汽车产业结构的同时，大力促进电子信息、高端装备制造、生物医药、绿色食品、新材料、新能源等新兴产业发展，打造过百亿元的产业集群。重点确保人福医药、华润生物、上善堂药业等项目投产见效，支持华昌达工业机器人、三助智能装备、斯堪维亚智能装备等项目建设，推进东风特汽新能源客车、新能源专用车研发生产和万润新能源材料产业基地建设，加快竹溪金铜岭、茅箭正发等电子园区建设。三是提升素质。营造尊重企业家、爱护企业家的社会氛围，打造开放开明、敢闯敢冒、守法守信、创业创新的企业家队伍。支持企业推进管理创新，建立健全现代企业制度，增强企业竞争力。

——筑牢基础性地位，发展现代农业。以工业化理念谋划农业、产业化方式开发农业，加快农业规模化、特色化、精深化发展，努力寻求农业发展方式新突破，开辟农业结构优化新途径，取得农民增收新成效，迈出美丽乡村建设新步伐。一是大力推进农业产业化。深入推进“四个百万”基地和农业标准化示范区建设，新增和改造基地35万亩，新增山羊出栏6万只。加快实施“四个一批”工程，着力推进8个农产品加工园区建设，新增规模以上农产品加工企业20家，实现农产品加工产值350亿元以上。突出打响武当道茶和房县黑木耳两大国字号品牌，力争新认证“三品一标”10个以上。二是创新农村经营机制。支持各类业主积极参与农业产业化经营，大力培植新型农村经营主体，新增市级合作社100家、家庭农场100家、专业大户1000家以上。推动农村土地（山场）资源向大户流转，发展适度规模经营。抓好国家“新型职业农民培育”整市整县推进试点工作，着力培育一批适应现代农业发展的职业化、专业化农民。三是提升农业可持续发展能力。加大农田水利、农村道路、农村环境综合整治等建设，改造县乡道路58公里，新建通村公路800公里；除险加固病险水库18座，解决农村安全饮水13.5万人。

——打造支撑性功能，繁荣现代服务业。坚持生产性、生活性服务业并举，传统、现代服务业并重，使服务业多点发力、提速增效。一是突破性发展新兴服务业。大力支持武当山五龙景区、太极湖、郧阳岛、子胥湖、大川生态旅游区、黄龙古镇等旅游项目群建设，全力推进城郊生态文化游憩带建设，着力打造美丽乡村游、城郊休闲游、汉江水上游、环库生态游等一批精品旅游线路，力争全年接待游客3900万人次，实现旅游收入280亿元。加快发展电商服务业，开工建设电商产业园，淘宝·特色中国·十堰馆正式运营，支持郧西涧池乡创建淘宝乡镇，打造湖北小蜜蜂、汽配人网等一批电商龙头企业。二是改造提升商贸流通业。重点支持三大物流园区和三大物流中心建设，加快推进万达广场、林安商贸物流城、昌升国际物流园、华西农商城等重点项目建设。积极推进社区商业综合服务中心建设，加快建设改造一批农产品批发市场、农贸市场。大力推进“武当珍品汇”在全国尤其是在北京、武汉的布点。力争社会消费品零售总额增长13.5%。三是拓展服务业发展空间。结合旧城改造，加快“退二进三”、“腾笼换鸟”和异地置换步伐，积极组织批发出城，零售进城，有序整合空间，高效配置资源。

——突出要素性引领，狠抓创新驱动。实施《秦巴山片区（十堰）科技创新中心建设规划》，积极创建国家可持续发展实验区和国家农业科技园。大力实施科技企业创业与培育工程、科技成果转化工程和能力提升工程，着力推动工业转型升级。新认定高新技术企业30家，完成技术交易8亿元，高新技术产业增加值增长15%。培育精品名牌，支持企业争创名牌产品和驰名商标。深入推进质量强市和标准化战略，推进十堰制造向十堰创造转变。

（四）扩大投资消费，培植增长拉力

　　投资是经济发展的驾辕之马，消费是稳定增长的重要基础。二者既是促进经济现实增长的引擎，也是夯实基础、增强后劲的底盘，必须着力把投资消费工作做细做实做牢。

——千方百计扩大有效投资。扎实开展项目建设竞进年、项目储备提质年、社会投资促进年活动，集中力量、大员上阵、全员行动抓项目、抓投资，确保全社会固定资产投资完成1300亿元以上。一是拓宽投资渠道。紧紧围绕高新技术产业、现代农业、现代服务业、环保、基础设施、城市地下管网改造等投资方向，牢牢抓住国家重点投资政策及向中西部倾斜的机遇，争取更多的投资落户十堰；创新投融资体制机制，探索推进政府和社会资本合作（PPP）等多种投资模式；鼓励和吸引民间投资；加快构建多层次资本市场，支持企业直接融资；争取民生、浦发银行落户十堰。二是推进投资落地。突出抓好150个市级重点项目建设，力保武十城际铁路、京能热电、夹河关水电站全面开工建设，环丹江口库区生态公路加快推进，武当山机场、火车站北广场、十竹路、316国道白浪段、黄龙段改造全面完工。三是抓好投资储备。精心编制“十三五”规划，科学谋划，认真筛选，储备一批事关全局、影响深远、带动力强的重大项目，挤进国家、省“十三五”规划笼子，构建“规划一批、储备一批、申报一批、建设一批、完成一批”的项目梯次推进体系。

——竭尽全力促进消费升级。进一步拓宽消费领域，改善消费环境，提供消费产品，激活消费需求。支持社会力量兴办养老、健身、健康、医疗等服务机构，满足多层次、多元化需求。加快实施养老、健康、家政信息、旅游休闲、住房、绿色生态、教育文体等七大领域消费工程，争创全国养老（家政）服务业示范城市。改善农村商品流通条件和消费环境，加强农村路网、电网、互联网的建设，挖掘消费潜力。#PAGE_BREAK#

　　（五）建设生态文明，增强十堰魅力

　　良好生态是最公平的公共产品和最普惠的民生福祉，也是十堰得天独厚的优势品牌。建设和保护好生态环境是我们天大的责任，要倍加珍惜绿色财富，坚定不移走生态文明之路，努力让山更绿、水更清、天更蓝、空气更清新，使美丽十堰绽放出更加迷人的魅力。

——扎实建设生态文明示范区。坚持绿色发展、生态崛起，强力推进生态建设，让生态成为十堰第一资源、第一品牌。精心组织“五城联创”，实施生态市规划、生态文明先行示范区三年行动计划，建设一批清洁型小流域、农业面源污染治理、库区地灾防治等示范工程，建成一批生态乡镇、生态村。创新完善生态补偿、节能环保、政绩考评等机制，提高生态文明指标在综合考评中的比重。加大国家森林城市创建力度，深入开展“绿满十堰”活动，以城区、丹江口市、堵河流域沿线为重点，推进造林绿化；全面完成武当山机场、高速公路、工业园区、十堰生态滨江新区等周边裸露山体生态修复任务，完成人工造林31.4万亩；加快实施“丹治”二期工程，加大库区水土保持治理力度。大力发展绿色产业，建设生态工业园区和再生资源基地，发展循环经济。支持郧阳区建设“水生态文明示范区”。

——全面优化空间布局。根据自然资源和生态环境承载能力，科学谋划空间开发格局，编制实施《十堰市主体功能区建设试点示范方案》，合理确定城镇、农业、生态三类空间比例，促进生产空间集约高效、生活空间宜居适度、生态空间山清水秀。全面推进生态红线管控，划定公布十堰市生态红线，加强南部生物多样性保育区、丹江口库区及沿岸水质保障区、中部丘陵水源涵养区、北部生态修复区等区域生态保护，以点状分布的禁止开发区域为重要组成，构建以山脉、水系为骨干，以江河、水库、山林、田地为基本生态要素的区域生态安全网络框架。

——切实加强环境保护。向污染宣战，既打攻坚战，又打持久战。坚持源头严防、过程严管、后果严惩，始终保持环境执法的高压态势。全面完成《丹库“十二五”规划》任务，确保环保设施全部达标运营。加快五河、城镇纳污河流污水收集管网、河道生态修复等工程建设，新建污水管网300公里，确保五河水质达标。加强对重点涉气企业环境监管，加大新能源汽车推广力度，执行黄标车、无标车城区禁行限行规定，确保按新标准全市空气质量达标率70%以上，集中式饮用水源水质达标率100%。

　　（六）推进新型城镇化，挖掘区域发展潜力

　　城镇化是现代化的必由之路，是破除城乡二元结构的重要依托。必须推进城乡统筹发展，走以人为本、“四化同步”、优化布局、生态文明、传承文化的新型城镇化道路，高标准做好城乡规划建设管理工作，努力让人民群众生活得更舒适。

——加快“一核多支点”建设步伐。立足高品质、创特色、建精品，强化规划引领，大力提升中心城区功能。启动建设京能热电铁路、高铁站广场、神鹰一路、石家沟路、京中路、大连路、吉林路等道路；加快完成林荫大道、机场大道、西城大道、风神大道三期、北环路、湖南路、襄阳路、源园路等续建道路；优化改造一批市政道路微循环。开工建设市客运和长岭公交换乘中心，完成火车站北广场建设。推进四方山生态公园建设，启动南水北调主题公园、牛头山森林公园、环库区绿道、十堰大道绿道等精品园林绿化项目建设。续建第三水厂及配套管网。完成天然气对接入户2万户。完善郧阳区规划，加快市、区规划、交通、产业、市场等深度融合；启动水堤沟至长岭公路建设，加快郧阳沧浪洲汉江大桥、郧府大道、郧阳大道建设，推进市人民医院滨江医院、湖北医药学院长岭校区建设。大力支持各县市道路交通、能源电力、通信网络、农田水利、公共服务等基础设施建设。大力发展县域经济，围绕做大基地、做强龙头、做优产品、做长链条、做响品牌，大力扶持郧阳区农产品加工、丹江口市水产业开发、郧西文化旅游、竹溪光电子、竹山绿色食品、房县生物医药等重点产业发展，力争县域生产总值增长12 %以上。

——着力推进以人为核心的新型城镇化。纵深推进“两带”建设。加快竹房城镇带建设，确保实现五年大变化。提高中心城镇承载力，重点抓好县城、中心镇以及中心社区功能的完善和拓展；强化产业支撑，大力发展商贸物流、乡村旅游业，推进农村劳动力就近转移；加快城乡一体公共服务体系建设，推进道路、电力、信息网络、商业网点等无缝对接。加强汉江生态经济带建设，以县城为中心，以集镇和片区为节点，推进沿江、沿路生态城镇体系建设。大力推进美丽乡村建设，加快11个国家级重点镇、8个全省重点中心镇、6个全省特色镇建设，抓好1个省级和7个市级“四化同步”示范乡镇建设，创建中国传统村落2个、省级宜居村庄14个，新建续建农村新社区20个、生态家园示范村110个，改造农村危房1万户以上。

　　（七）保障改善民生，增进社会和力

　　民惟邦本。保障和改善民生是我们一切工作的出发点和落脚点，必须切实做好民生工作，努力建设幸福十堰。

——竭力加强民生保障。坚持守住底线、突出重点、保障基本，倾注精力、集中财力解决民生问题。多渠道开发就业岗位，大力促进创业带动就业。全面实施机关事业单位养老保险制度改革，继续扩大社会保险覆盖面，不断提高统筹层次和保障水平。加快推进市第二老年公寓建设，新改建农村福利院16所、城乡社区养老服务中心80个、社区服务站15个，改造五保危房3000户。加强各项救助政策衔接和落实。落实库区移民后期扶持政策，加强地质灾害监测防治，确保移民安稳致富。加大住房保障力度，实施老旧小区、城中村、棚户区综合改造。围绕“四双”扎实开展精准扶贫，做好安全饮水、特色产业增收、村村通客车等重点惠民解困工作，深入实施贫困村整村推进，完成雨露计划培训1万人，生态扶贫搬迁1万户，减少贫困人口9万人，促进贫困群体同全市人民一道奔小康，尽力阻断贫困代际传递。

——全面发展社会事业。坚持立德树人，全面推进素质教育，均衡发展义务教育，加快发展职业教育，扩大普惠性学前教育，逐步提高特殊教育水平；进一步优化城区学校布局，加快十堰高级职业学校、车城高中、柳林中学、北京路中学、重庆路小学、龙门小学6所学校迁建新建和8所思源学校援建工作，大力支持高校发展。传承和弘扬地方特色文化，加大文物和非物质文化遗产保护力度，培育和规范文化市场，提升文化产业发展水平。推动全民健身运动，强化公共体育设施建设，新建农民体育健身工程60个、健身路径30条。全面推进农村广播电视户户通和乡镇影院建设。加快县级综合档案馆建设。深化医药卫生体制改革，推进县级公立医院综合改革和分级诊疗试点，支持社会办医；巩固新农合制度，认真执行重大疾病补偿和疾病应急救助政策；强化公共卫生能力保障，支持市妇幼保健院迁建等卫生项目建设。继续稳定低生育水平，综合整治出生人口性别比，不断提升计生优质服务水平。

——大力创新社会治理。深入推进平安十堰建设，开展严打专项整治。加强人防、巩固物防、提升技防，推进天网、地网、光网建设，继续加强城乡网格化建设，构筑立体化治安防控体系。大力开展法治宣传教育，推进人民调解规范化建设，做好“六五”普法迎检工作。高度重视信访工作，扎实开展矛盾纠纷“大排查、大调处”活动。时刻紧绷安全生产之弦，严格执行安全生产法律法规，全面落实责任制，坚决遏制重特大安全事故发生。严厉打击制售假劣食品药品行为，深入开展餐桌污染专项治理，用更严的监督、更严的处罚、更严的问责确保食品药品安全。大力开展全民国防教育，扎实做好优抚安置、涉军维权稳控等工作，积极争创全国双拥模范城七连冠和全省双拥模范城八连冠。

——深入建设精神文明。积极开展“中国梦”宣传教育，培育和践行社会主义核心价值观，加强公民道德和精神文明建设。扎实做好国家、省文明城市，文明村镇，文明单位创建工作。认真开展道德模范、十堰好人等评选。全面推进政务诚信、商务诚信、社会诚信和司法公信建设，培养良好的社会风尚。

（八）加强自身建设，提高行政能力

　　新常态需要新作为，新作为呼唤新作风。各级政府及工作人员为党尽职、为人民履责，必须牢记使命，不负重托，从方方面面把握，从点点滴滴做起，显公仆本色，让人民满意。

——做到心中有党，忠诚磊落。为政之道、谋事以忠，我们必须把忠诚作为天职，对党忠诚，对国家忠诚，对人民忠诚，对事业忠诚，把牢政治方向，坚定政治信念，维护党的权威，增进党的团结，与党中央保持高度一致，坚决贯彻中央、省委、市委决策部署。在党言党，在党为党，在党忧党，以身许党许国，以身报党报国。要境界高远，襟怀坦荡，矢志为公，不怀私念，不计私怨，不谋私利，言行一致，表里如一，让党放心。

——做到心中有民，勤政敬业。心中始终装着人民，先天下之忧而忧，后天下之乐而乐，以民之所好为好、民之所恶为恶，把人民的期盼作为最高追求，把人民的利益摆在最高位置，做到民有所呼我有所应，民有所求我有所为，民有所盼我有所办。不辞辛劳、不避疲劳、不恤苦劳，聚全部心智，献所有精力，夙夜为公，勤勉工作，为人民甘于呕心沥血，为事业不惜鞠躬尽瘁。

——做到心中有法，秉公持正。强化宪法意识和法治理念，尊法、学法、守法、用法，建设法治政府、法治社会。定准位，站好位，不越位，不错位，不缺位，做到位。坚决执行人大及其常委会决议决定，自觉接受市人大及其常委会的法律监督、工作监督和政协的民主监督，认真办理人大代表建议和政协委员提案，广泛听取民主党派、工商联、无党派人士和人民团体意见，虚心接受社会监督和舆论监督。坚持依法科学民主决策，完善重大决策风险评估、合法性审查等机制。提高行政执法水平，完善行政复议制度。扎实推进百姓问政，深入开展政务公开，创造条件让人民监督政府。深入推进依法行政，依法严惩各类违法行为，加大关系群众切身利益的重要领域执法力度，确保有法必依、执法必严、违法必究。强化行政问责追责力度，坚决纠正行政不作为、慢作为、乱作为，严厉惩处失职、渎职行为。

——做到心中有责，务实担当。视责如山，守责如城，履责如冰，切实做到责随职走，心随责定，勇于担责，敢于负责。面对困难不退缩，遇到矛盾不回避，不做太平官，不当“甩手掌柜”，以只想当官不想干事、只想揽权不想担责、只想出彩不想出力为耻，始终保持干事创业的热情、动真碰硬的豪情、奋发有为的激情，不断创造经得起实践、群众和历史检验的业绩。坚持实干实为、善做善成，力戒夸夸其谈、坐而论道，拿出招招求实、步步踏实的实劲，肯干苦干、百折不挠的韧劲，不达目的誓不罢休的拼劲，不避险不畏屈不怕事的“苕”劲，为十堰的发展拼搏奋斗。

——做到心中有戒，清廉守矩。把政府行为置于灿烂的阳光下，把手中权力关进制度的“铁笼子”，把自己言行与纪律规矩对表，始终敬畏手中的权力，敬重服务的人民，依法用权，秉公用权，廉洁用权，心有所畏，言有所戒，行有所止。严守政治纪律、政治规矩，严守组织纪律、财经纪律，严守廉政准则、行为规范，坚决执行“八项规定”，自觉践行“三严三实”，驰而不息纠正“四风”，持之以恒改进作风，切实做到廉洁从政，清白做人，干净干事，不越制度“底线”，不碰纪律“高压线”。严格落实政府系统党风廉政建设的主体责任，认真履行“一岗双责”，加强审计监督、廉政监察和效能监察，严肃查处各类腐败行为，坚定不移推进党风廉政建设和反腐败斗争。

各位代表！今年将继续办好十件实事：一是建设标准化村卫生室240个；二是建设“放心粮油”配送中心9家、连锁店155家；三是建设绿色环保公交线路6条；四是改造老旧小区6个，建设社区养老服务中心30个、果蔬便民门店50家；五是改造城区微循环道路10条，垃圾中转站10座；六是实施城区中小学幼儿园门前交通畅通工程；七是完成有线电视和宽带网络“村村通”500个，智能广播“村村响”全覆盖；八是完成农村低压电改造4.8万户；九是实施85个扶贫重点村整村推进，创建生态乡镇、生态村270个；十是城镇新增就业4万人，创业带动就业2万人。

各位代表！新常态送我们站在了历史的新起点，新机遇助我们迈上了发展的新征程。让我们万众一心，同心同德，在市委的坚强领导下，始终保持锐意开拓的气场不变、竞进提质的劲头不松、转型跨越的力度不减、干事创业的激情不泄，顺势而为，乘势而上，实现不负美好时代的新作为，奋力开创生态文明建设、转型跨越发展走在全省前列和区域性中心城市建设的新局面！
